# Supplementary material for: Linear regression reporting practices for health researchers, a cross-sectional meta-research study
Source: PLoS One. 2025 Mar 20;20(3):e0305150. doi: 10.1371/journal.pone.0305150 (PMC11925299; doi:10.1371/journal.pone.0305150)
Supplement: S1 Table — (DOCX) [file pone.0305150.s001.docx]

S1 Table: Full reporting of agreement and reliability for statistical raters for 95 papers.

| Variable | Agreement | Pe | Gwet | 95% CI | SE | p-value |
| --- | --- | --- | --- | --- | --- | --- |
| **Rating 1 vs Rating 2** |  |  |  |  |  |  |
| Coefficient | 85% | 33% | 0.78 | 0.66, 0.90 | 0.06 | <0.001 |
| Confidence intervals | 85% | 50% | 0.71 | 0.56, 0.85 | 0.07 | <0.001 |
| Standard error | 86% | 36% | 0.79 | 0.66, 0.91 | 0.06 | <0.001 |
| R-Squared | 82% | 49% | 0.65 | 0.50, 0.81 | 0.08 | <0.001 |
| F/t statistics | 87% | 28% | 0.82 | 0.72, 0.93 | 0.05 | <0.001 |
| Degrees of freedom | 92% | 8% | 0.91 | 0.84, 0.98 | 0.03 | <0.001 |
| N in models | 57% | 49% | 0.16 | -0.05, 0.37 | 0.11 | 0.131 |
| Direction interpreted | 72% | 49% | 0.44 | 0.26, 0.63 | 0.09 | <0.001 |
| Size interpreted | 65% | 39% | 0.43 | 0.23, 0.62 | 0.10 | <0.001 |
| p-values | 75% | 24% | 0.67 | 0.54, 0.79 | 0.06 | <0.001 |
| Importance of parameters | 48% | 30% | 0.27 | 0.11, 0.42 | 0.08 | 0.001 |
| Collinearity evaluated | 61% | 29% | 0.45 | 0.30, 0.59 | 0.07 | <0.001 |
| variables transformed | 69% | 21% | 0.61 | 0.48, 0.75 | 0.07 | <0.001 |
| Scaled appropriately | 40% | 21% | 0.24 | 0.11, 0.38 | 0.07 | <0.001 |
| Process variable selection | 37% | 16% | 0.25 | 0.13, 0.36 | 0.06 | <0.001 |
| Variable selection strategy | 92% | 3% | 0.91 | 0.85, 0.97 | 0.03 | <0.001 |
| Model significance criteria | 91% | 6% | 0.90 | 0.83, 0.97 | 0.03 | <0.001 |
| **Rating 1 vs Prevalence** |  |  |  |  |  |  |
| Coefficient | 94% | 31% | 0.91 | 0.83, 0.98 | 0.04 | <0.001 |
| Confidence intervals | 88% | 50% | 0.77 | 0.64, 0.90 | 0.07 | <0.001 |
| Standard error | 92% | 32% | 0.88 | 0.79, 0.96 | 0.04 | <0.001 |
| R-Squared | 89% | 49% | 0.79 | 0.67, 0.92 | 0.06 | <0.001 |
| F/t statistics | 92% | 27% | 0.89 | 0.80, 0.97 | 0.04 | <0.001 |
| Degrees of freedom | 96% | 12% | 0.95 | 0.90, 1.00 | 0.02 | <0.001 |
| N in models | 74% | 49% | 0.49 | 0.31, 0.67 | 0.09 | <0.001 |
| Direction interpreted | 80% | 46% | 0.63 | 0.47, 0.79 | 0.08 | <0.001 |
| Size interpreted | 76% | 36% | 0.62 | 0.46, 0.78 | 0.08 | <0.001 |
| p-values | 86% | 23% | 0.82 | 0.73, 0.92 | 0.05 | <0.001 |
| Importance of parameters | 66% | 26% | 0.54 | 0.40, 0.69 | 0.07 | <0.001 |
| Collinearity evaluated | 77% | 28% | 0.68 | 0.56, 0.80 | 0.06 | <0.001 |
| variables transformed | 78% | 22% | 0.72 | 0.60, 0.83 | 0.06 | <0.001 |
| Scaled appropriately | 52% | 20% | 0.39 | 0.25, 0.53 | 0.07 | <0.001 |
| Process variable selection | 59% | 16% | 0.51 | 0.39, 0.63 | 0.06 | <0.001 |
| Variable selection strategy | 94% | 4% | 0.93 | 0.88, 0.99 | 0.03 | <0.001 |
| Model significance criteria | 92% | 11% | 0.91 | 0.84, 0.97 | 0.03 | <0.001 |
| **Rating 2 vs Prevalence** |  |  |  |  |  |  |
| Coefficient | 92% | 33% | 0.87 | 0.78, 0.96 | 0.05 | <0.001 |
| Confidence intervals | 88% | 50% | 0.77 | 0.64, 0.90 | 0.07 | <0.001 |
| Standard error | 91% | 36% | 0.85 | 0.75, 0.95 | 0.05 | <0.001 |
| R-Squared | 84% | 48% | 0.70 | 0.55, 0.84 | 0.07 | <0.001 |
| F/t statistics | 89% | 29% | 0.85 | 0.75, 0.95 | 0.05 | <0.001 |
| Degrees of freedom | 92% | 12% | 0.90 | 0.84, 0.97 | 0.04 | <0.001 |
| N in models | 71% | 50% | 0.41 | 0.22, 0.60 | 0.09 | <0.001 |
| Direction interpreted | 77% | 48% | 0.55 | 0.38, 0.72 | 0.09 | <0.001 |
| Size interpreted | 73% | 39% | 0.55 | 0.38, 0.73 | 0.09 | <0.001 |
| p-values | 83% | 22% | 0.78 | 0.68, 0.89 | 0.05 | <0.001 |
| Importance of parameters | 58% | 27% | 0.42 | 0.27, 0.57 | 0.08 | <0.001 |
| Collinearity evaluated | 72% | 28% | 0.60 | 0.47, 0.74 | 0.07 | <0.001 |
| variables transformed | 78% | 23% | 0.71 | 0.59, 0.83 | 0.06 | <0.001 |
| Scaled appropriately | 46% | 22% | 0.32 | 0.18, 0.45 | 0.07 | <0.001 |
| Process variable selection | 48% | 16% | 0.39 | 0.26, 0.51 | 0.06 | <0.001 |
| Variable selection strategy | 89% | 6% | 0.89 | 0.82, 0.96 | 0.03 | <0.001 |
| Model significance criteria | 91% | 8% | 0.90 | 0.83, 0.96 | 0.03 | <0.001 |

Agreement = Observed agreement, Pe = The expected agreement by chance, Gwet = Gwet agreement coefficient, 95% CI = Gwet 95% confidence intervals, SE = Standard Error. Variables were either binary or nominal and did not require weighting.
