# Supplementary material for: Linear regression reporting practices for health researchers, a cross-sectional meta-research study
Source: PLoS One. 2025 Mar 20;20(3):e0305150. doi: 10.1371/journal.pone.0305150 (PMC11925299; doi:10.1371/journal.pone.0305150)
Supplement: Attachment — Submitted filename: Response to Reviewers.docx [file pone.0305150.s002.docx]

| **Comments from editor**  Your manuscript has been reviewed and requires modifications prior to making a decision. The comments of the reviewers are included at the bottom of this letter. The manuscript presents a well-conducted study that underscores the importance of statistical methods and transparent research reporting. While the study is valuable, some improvements are recommended: the theoretical aspects of regression could be shortened, as they are widely available in standard texts, and the role of statistics in health systems could be elaborated to provide better context. Additionally, the authors should clarify certain methodological choices, such as the selection of specific timeframes, and consider emphasizing the need for involving statisticians with relevant expertise throughout research projects to enhance rigor and reliability. These adjustments will further refine the manuscript and improve its clarity for readers. | Thank you for the opportunity to make corrections to this paper. See our response to the reviewers' comments below. |
| --- | --- |
| 1. Please ensure that your manuscript meets PLOS ONE's style requirements, including those for file naming. The PLOS ONE style templates can be found at  [https://journals.plos.org/plosone/s/file?id=wjVg/PLOSOne_formatting_sample_main_body.pdf](https://urldefense.com/v3/__https:/journals.plos.org/plosone/s/file?id=wjVg*PLOSOne_formatting_sample_main_body.pdf__;Lw!!NVzLfOphnbDXSw!GU4WdvIRKIdm7zUEd7uHKkKnsxAdJETE6mJjvyoqfNJJuCxYBGBEp4YUN0gDcc6DwHNMYM7umdT_Gt0TggOu$) and  [https://journals.plos.org/plosone/s/file?id=ba62/PLOSOne_formatting_sample_title_authors_affiliations.pdf](https://urldefense.com/v3/__https:/journals.plos.org/plosone/s/file?id=ba62*PLOSOne_formatting_sample_title_authors_affiliations.pdf__;Lw!!NVzLfOphnbDXSw!GU4WdvIRKIdm7zUEd7uHKkKnsxAdJETE6mJjvyoqfNJJuCxYBGBEp4YUN0gDcc6DwHNMYM7umdT_Gr5VXHZJ$) | We have followed the style guide |
| **Funding**  2. Please note that funding information should not appear in any section or other areas of your manuscript. We will only publish funding information present in the Funding Statement section of the online submission form. Please remove any funding-related text from the manuscript. | The funding statement has been removed from the manuscript. |
| 3. Thank you for stating the following financial disclosure:  "There was no cost associated with this research except for attending conferences. These costs were covered by the primary author’s PhD allocation from the health faculty, Queensland University of Technology, and scholarships. The Statistical Society of Australia (SSA) and the Association for Interdisciplinary Meta-research & Open Science (AIMOS) supported the primary author with travel grants to attend their respective conferences. These scholarships did not influence the results of the study."    Please state what role the funders took in the study.  If the funders had no role, please state: "The funders had no role in study design, data collection and analysis, decision to publish, or preparation of the manuscript."  If this statement is not correct you must amend it as needed.  Please include this amended Role of Funder statement in your cover letter; we will change the online submission form on your behalf. | Please see the updated funding statement.  There was no cost associated with this research except for attending conferences. These costs were covered by the primary author’s PhD allocation from the health faculty, Queensland University of Technology, and scholarships. The Statistical Society of Australia (SSA) and the Association for Interdisciplinary Meta-research & Open Science (AIMOS) supported the primary author with travel grants to attend their respective conferences. The funders had no role in study design, data collection and analysis, decision to publish, or preparation of the manuscript. |
| **References**  4. Please review your reference list to ensure that it is complete and correct. If you have cited papers that have been retracted, please include the rationale for doing so in the manuscript text, or remove these references and replace them with relevant current references. Any changes to the reference list should be mentioned in the rebuttal letter that accompanies your revised manuscript. If you need to cite a retracted article, indicate the article’s retracted status in the References list and also include a citation and full reference for the retraction notice. | Eight references have been added to the manuscript in response to reviewer comments. These can be seen in the bib file and include Zhu, Y., et al. (2024), Karimi-Maleh, H., et al. (2023), Tikka, et al. (2021), Moberg, J. et al. (2018), Prospective Studies Collaboration. (2002), Thelwall, M. (2024), Böschen, I. (2024), Statistical Society of Australia 2019.  One reference was also updated Goodman, S. (2008). |
| **Review Comments to the Author** |  |
| Reviewer #1: A very well drafted manuscript by the authors. In the article they have clearly explained how the statistical methods were poorly reported without sufficient details to reproduce them and also how to improve the statistical quality and direct health funding to effective treatments. | We thank you for your review. |
| Reviewer #2: It is an interesting study and underlines the importance of statistical test and the method of reporting the test and its output. However there are concerns which need to be addressed. 1. The authors can shorten the theoretical aspects of regression which can be obtained in standard statistical books. | We understand the reviewer's concern but feel the statistical theory adds value to the paper. This paper has been developed for two distinct audiences: health researchers and meta-researchers.  While this content can be found in textbooks, researchers increasingly rely on easy-to-access online content. One way to address poor statistical quality is to have accessible examples of how to report statistical information that contains accurate information and highlights what and why good reporting is important. One of the weaknesses of the SAMPL guidelines is that it is a list and does not show examples.  With this in mind, we have added a table with an example of good reporting for linear regression coefficients.  The other main audience is meta-researchers and statisticians who are more interested in the prevalence of poor reporting and the discussion around strategies for improvement. We have created clear subheadings that make it easy for the reader to skip sections they are not interested in.  **Added example**  “Good reporting practice involves not only presenting the numbers but also interpreting the results, contextualising their importance, and addressing why they matter. Below (Table 1 and 2) is an example of the write-up and coefficients table for good reporting of the simulated data presented in Fig 1.  Blood pressure was found to be associated with age (F(1, 198) = 262.3, p < 0.001), with blood pressure increasing by 1.03 mmHg (95% CI: 0.91, 1.16) per year or 10.3mmHg as age increases by 10 years (Table 2). Age explained approximately 57% (R^2^ = 0.57) of the variance in blood pressure. Even small increases in blood pressure can have a significant impact on cardiovascular health, as sustained elevations contribute to an increased risk of adverse outcomes such as heart attack and stroke. For example, in middle age, a 2 mmHg increase in systolic blood pressure is associated with a 10% higher risk of ischemic heart disease and a 7% higher risk of stroke [34]. “ |
| Reviewer #3: Excellent piece of work. Just a couple of minor comments.  1. You only know what is *reported*. You say "Authors rarely check multicollinearity...". Probably true, but I know I (almost) always check multicollinearity but I don't think I have ever mentioned it in a publication. | We agree this was an oversight, and we have updated the wording in the multicollinearity paragraph that compares the results to other research to clarify that we are talking about reporting. |
| 2. PLOS ONE has no word limits, so authors should be able to report everything needed. However, other medical journals often have very strict word limits, and some still do not allow supplementary material. People learn to leave things out to fit word limits, and I think this should be mentioned. It is part of the system-wide problem. | Thank you for your suggestion. We agree that this is an important point and have added a section to the system-wide problem.  “There are also structural issues with journals focusing on word and table limits rather than good reporting, with poor reporting reinforced by journals requiring short statistical sections rather than comprehensive and transparent reporting. Our study highlighted that even when there are no word limits, statistical sections are often not reported in enough detail to reproduce, suggesting that this may be a learned behaviour.” |
| Reviewer #4: As a strong supporter of greater transparency and improved quality in research reporting, I welcome and appreciate meta-research studies that emphasise the need for higher research quality and better reporting. I believe this study is a valuable contribution to this area, and I commend the authors for their appropriately designed and conducted work. The study methods are quite straightforward, and I don’t have any major remarks. I have only a few comments, which I hope will help make the article clearer for readers and suggest a few points the authors may want to consider. | We thank you for your feedback |
| **INTRODUCTION** |  |
| [Comment #1] In the introduction (page 2), the author begins the first paragraph with: “Health systems are generally complex and often comprise a network of interrelated variables. Statistical methods can help untangle and understand these relationships, allowing the quantification and estimation of the effects of diseases and treatments.” This opening sentence is somewhat unclear. Health systems utilise results from studies on the effects of diseases and treatment effectiveness to inform decision-making. However, these decisions are also influenced by factors other than statistics (e.g., see the GRADE Evidence-to-Decision framework). While the role of statistics is unquestionably important, it is not the only factor. I suggest elaborating this a bit more, clarifying the role of statistics in health system decisions and policy, or changing the starting sentence. As presented, it seems that regression models are directly linked to uncovering the complexity of the healthcare system, whereas in the context of this study, they mainly refer to the statistical results of studies.  Moberg, J., Oxman, A.D., Rosenbaum, S. et al. The GRADE Evidence to Decision (EtD) framework for health system and public health decisions. Health Res Policy Sys 16, 45 (2018). [https://doi.org/10.1186/s12961-018-0320-2](https://urldefense.com/v3/__https:/doi.org/10.1186/s12961-018-0320-2__;!!NVzLfOphnbDXSw!GU4WdvIRKIdm7zUEd7uHKkKnsxAdJETE6mJjvyoqfNJJuCxYBGBEp4YUN0gDcc6DwHNMYM7umdT_GvEy-Neu$) | We appreciate the feedback and agree that the introduction can better situate the role of statistical methods within the broader decision-making process.  “Health systems are inherently complex, requiring decisions that balance scientific evidence with practical considerations such as feasibility, equity, and stakeholder perspectives [1]. Central to this process is the role of statistics, which aid in the  interpretation of study results to provide the quantitative evidence necessary for informed decision-making about the effects of diseases and treatments.” |
| [Comment #2] It may be worth mentioning that the quality of reporting is also important for assessing the risk of bias. Without sufficient information, the assessibility of the risk of bias is reduced.  Tikka C, Verbeek J, Ijaz S, et al. Quality of reporting and risk of bias: a review of randomised trials in occupational health. Occupational and Environmental Medicine 2021;78:691-696. | This has now been included in the introduction in the paragraph discussing reporting guidelines.  “Reporting quality is crucial for assessing the risk of bias. Insufficient information reduces the ability to evaluate bias, undermining the credibility and reliability of the study’s findings and limiting their  value for guiding clinical practice or informing policy decisions [15]. |
| **METHODS** |  |
| - Sampling (randomisation)  [Comment #3] It is unclear why 2019 was selected for the search. Could the authors elaborate a bit more on this decision? This choice can have implication of the generalisability of the findings. This should be probably also addressed in the discussion/limitation. | We have explained why we chose 2019 and added a section to the limitations.  **Randomisation**  “This study was conducted in 2020/21, and 2019 was chosen to gain a current snapshot of reporting practices. While not planned, this research coincided with the full year before the COVID-19 pandemic, avoiding any disruption that may have been caused by changes in the research environment due to the shutdowns [29].”  **Limitations**  “To obtain stable estimates given the low prevalence of some reporting behaviours, the sample was made in a single year rather than spanning multiple years. While this approach may limit generalisability, the study's findings align consistently with existing literature on researchers reporting statistical results.” |
| [Comment #4] I cancelled some comments and requests for clarification after realising they were addressed in the medRxiv preprint. It seems this paper presents a shorter version of the method section from the preprint. I suggest mentioning this (e.g., that more details about the methods are available in the preprint) at the beginning of the methods section. However, I wonder why these details cannot be included in the current paper, as it might be more convenient for readers. Just a personal opinion. | This is the second paper in a series of four papers that will be the basis of the first author's PhD. The first paper, the pre-print referred to <https://www.medrxiv.org/content/10.1101/2024.02.15.24302870v1>  is still under review and at PLOS ONE (submitted in February), so the papers will likely be published out of order. An effort was made to write this paper to be self-contained while avoiding self-plagiarism. We have made the pre-print citation more prominent at the top of the methods. The original protocol is also available on Github: <https://github.com/Lee-V-Jones/statistical-quality>. |
| **Comparison of results to other research** |  |
| [Comment #4] The paper by Strasak et al., referred to articles published before SAMPL was available. This could be mentioned and considered. | We have added the timing of the SAMPL in the Comparison of results to other research section.  “While the study by Strasak et al. [55]  occurred before the publication SAMPL guidelines in 2013 [17], Fernandez–Nino and  Hernandaz–Montes [56] assessed 108 papers from 2000 to 2017 in three six-year periods, finding an increased volume of reported regressions, with most items on their checklist worsening over time. Our study also showed a high prevalence of poor reporting, indicating that introducing guidelines like SAMPL without enforcement does  not resolve poor reporting issues.” |
| - Multicollinearity [Comment #5] The study did not differentiate whether the regressions were used for prediction/forecasting or causal inference. Multicollinearity is considered more problematic for (causal) inference than prediction. This may be discussed. | We have included this point in the Comparison of results to other research in the multicollinearity section.  “While our study did not distinguish whether the regressions were conducted for prediction or statistical inference, this is an important distinction because the implications of multicollinearity depend on the analysis objective. In causal/associational models, multicollinearity can distort coefficient estimates and hinder their interpretation, whereas prediction models  prioritise overall accuracy and may accommodate correlated predictors if predictive  performance remains unaffected.” |
| **RESULTS AND DISCUSSION** |  |
| - System-wide reform of statistical practices [Comment #6] This is more of a comment and not necessarily something the authors need to include in their article. However, I believe it should be emphasised more that the academic environment provides little incentive for high-quality research, where involving a statistician would be a consequential/logical step for conducting more rigorous research. Academia continues to prioritise quantity over quality for various reasons. While efforts such as sensitisation, the Social Ecological Model, or other initiatives like the Hong Kong Principles or CoARA are commendable, they do not appear to be effective. Only structural changes and enforced measures are likely to improve the situation. Maybe reputable national institutions such as the Statistical Society of Australia can press academic and governmental institution to implement (i.e., making mandatory) higher standards. | Thank you for sharing your observations. We agree that the academic environment prioritises quantity over quality, which can hinder the adoption of rigorous research practices, including the involvement of statisticians. We share your concern that intervention without more substantial structural changes may have limited benefits. This is why we advocate the ecological model, which includes organisational and policy levels.  In Australia, large structural changes are required in how research is funded. The government does not fully fund research and relies heavily on university cross-funding research from teaching, philanthropic contributions, and researchers to cover costs. This funding gap poses significant challenges for research institutions and contributes to job insecurity. While a full discussion of these challenges was out of the scope of the paper, we did discuss diverting 1% of publishers’ profits and scientific funding to quality control, which can only be achieved nationally and requires federal government funding.  However, it’s also important to acknowledge that meaningful change in such a complex system requires a multifaceted approach and is often incremental. Cultural change is a gradual process built through consistent advocacy, education, and showcasing the long-term benefits of high-quality research. As we highlighted, there is a danger of the bystander approach, where everyone expects others to act rather than improve what is in their space. As statisticians, we educate researchers on the best statistical practices, as seen in this paper. We also believe journals and institutions could and should be doing more to improve reporting practices. |
| - Checklists and automated reviews  [Comment 7] I completely agree with what is presented by the authors, including the suggestion that journals should require reporting the full text, not just the page, in quality of reporting checklists. The authors also mention text-mining-based programs (statcheck), which unfortunately do not work well. However, the use of LLMs could be useful and well-suited for this purpose. The authors may want to emphasise that LLMs could facilitate the development of automatic tools in the future. This has also been suggested by Böschen, who criticised statcheck: [https://arxiv.org/abs/2408.07948](https://urldefense.com/v3/__https:/arxiv.org/abs/2408.07948__;!!NVzLfOphnbDXSw!GU4WdvIRKIdm7zUEd7uHKkKnsxAdJETE6mJjvyoqfNJJuCxYBGBEp4YUN0gDcc6DwHNMYM7umdT_GvF7ij1c$) [https://doi.org/10.48550/arXiv.2408.07948](https://urldefense.com/v3/__https:/doi.org/10.48550/arXiv.2408.07948__;!!NVzLfOphnbDXSw!GU4WdvIRKIdm7zUEd7uHKkKnsxAdJETE6mJjvyoqfNJJuCxYBGBEp4YUN0gDcc6DwHNMYM7umdT_Gv8ahnTV$) | We welcome the opportunity to discuss LLMs in the context of our study.  "with Böschen [79] reporting that Statcheck should not be used to detect irregularities in statistical results as it is unable to deal with even small  deviations in formatting. The current study found results were often incomplete and  inconsistently formatted, reflecting differing reporting practices across health fields, a  current barrier to automation.  “Zhu et al. [80] recently tried to establish if Large Language Models (LLMs) are as  good as statisticians. The authors used 11,623 examples to evaluate LLMs’ proficiency in specialised statistical tasks, including identifying appropriate statistical methods and parameters. The authors found that postgraduate statistical students had an accuracy of 53% when performing statistical tasks, whereas GPT-4o outperformed other LLMs and humans with an accuracy of 65%. The authors found that LLMs are good at  distinguishing different statistical tasks but may struggle to use domain knowledge, whereas humans are prone to task confusion; they recommended complementary use of  LLMs with researchers [80].  Our study did not compare large language models to statistical reviewers. Instead, each paper received two statistical reviews which were compared to assess reliability. Statistical reviewers were highly reliable in identifying common statistics but less reliable for questions requiring interpretation. While not quantitatively measured, the primary author reviewed all papers and every disagreement and noted when there seemed to be an apparent reason for disagreement. These reasons can be broken into four areas: Concentration, Task confusion, Experience, and Paper Coherence.  LLMs are expected to outperform reviewers when errors are related to concentration, such as missing statistical information in the text, but have been found to have low accuracy when reviewing becomes more complex [81]. Some papers had very poor statistical reporting and were challenging to review, often leading statisticians to interpret their best guesses based on what the authors reported. Some methods sections were unclear and may not match what was reported in the results, and often had tables without in-depth interpretation or identification of statistical tests used. This may also be challenging for LLMs as statisticians use broad contextual knowledge developed over their careers to interpret statistical methods and can identify different mistakes commonly made depending on the subject area and statistical packages used. Future research should consider codifying this knowledge to enhance the performance of large language models. Therefore, while automated tools are helpful, further development is required to increase accuracy before being used to review papers [81], and should only be used to aid the reviewer, such as helping screen the paper for checklists. Reviewers can then use this information to improve the interpretation of results [77]. |
| - Involvement of statisticians  [Comment 8] I agree with the authors' main arguments and their encouragement to involve statisticians in research projects. However, it is important to emphasise that not all statisticians possess the necessary expertise for every project (i.e., it is not enough to involve a statistician to tick the box). Specific areas of expertise should be considered when involving experts. For example, as mentioned earlier, developing prognostic models or structural causal models requires statisticians with different areas of expertise and contextual knowledge. As an editor, I have observed cases where involving statisticians did not generate better results because the statisticians lacked relevant specific knowledge. While the authors may find this point obvious, I strongly believe it is not generally recognised. Highlighting this could be valuable. | Thank you for the opportunity to highlight this point.  "Involvement of statisticians should not be a tick box exercise, as it is crucial to engage statisticians with the appropriate expertise and contextual knowledge to meet the specific demands of research projects. For instance, developing prognostic models or structural causal models requires specialised skills that not all statisticians possess. It is recommended, when possible, to use accredited statisticians, as professional bodies such as the Statistical Society of Australia bind members to a code of conduct that encourages them to offer services only within their professional competence and refrain from claiming expertise they do not possess. These guidelines support ethical practice and help researchers access high-quality statistical expertise, enhancing the reliability and rigour of research [87]. " |
| - Conclusions  [Comment #9] Related to my previous comments, I would modify “allowing statisticians and other methodological experts to be involved through the entire project cycle” to something like: “allowing QUALIFIED statisticians and other methodological experts in the specific area of investigation to be involved throughout the entire project cycle” | We agree and have changed to  “allowing qualified statisticians and other methodological experts to be involved through the entire project cycle” |
